# Can Antidromic and Orthodromic Stimulation Both Be Used for Correct Carpal Tunnel Syndrome Staging by J. D. Bland and L. Padua?

**DOI:** 10.3390/medicina61050938

**Published:** 2025-05-21

**Authors:** Vlada Meļņikova, Maksims Timčenko, Solvita Bērziņa, Guntis Karelis

**Affiliations:** 1 Graduate Medical Training, Rīga Stradiņš University, Dzirciema Street 16, LV-1007 Riga, Latvia; 2 Riga East University Hospital, Hipokrata Street 2, LV-1038 Riga, Latvia; guntis.karelis@aslimnica.lv; 3 P. Stradins Clinical University Hospital, Pilsonu Street 13, LV-1002 Riga, Latvia; maksims.timcenko@inbox.lv (M.T.); solbe@inbox.lv (S.B.); 4 Department of Infectology, Rīga Stradiņš University, Dzirciema Street 16, LV-1007 Riga, Latvia

**Keywords:** carpal tunnel syndrome, severe stage, antidromic and orthodromic stimulation

## Abstract

*Background and Objectives*: Padua (1997) and Bland (2000) have already proposed neurophysiological classification scales for patients with carpal tunnel syndrome (CTS), where the absence of orthodromic sensory response is used as a criterion of a severe stage. We hypothesized that antidromic values cannot be used equally for correct staging. *Materials and Methods*: We performed a consecutive investigation with nerve conduction studies in 60 arms of patients with CTS and prolonged distal motor latency. *Results*: In 11 out of 60 arms (18.3% of cases), orthodromic sensory nerve action potential (SNAP) was undetectable, while the antidromic SNAP was present. ROC curve analysis with Yoden index calculation were utilized in the study. The cut-off value of antidromic SNAP amplitude as a diagnostic marker of unrecordable orthodromic SNAP was 3.9 µV with high sensitivity and specificity. *Conclusions*: Our findings conflict with Padua et al.’s assertion that CTS staging can be determined irrespective of the stimulation technique. Antidromic SNAP amplitude is the most reliable parameter for predicting the absent orthodromic SNAP. Our study addresses the bias associated with the application of antidromic stimulation of median nerve sensory fibers for accurately staging moderate to severe CTS.

## 1. Introduction

Nowadays, carpal tunnel syndrome (CTS) is highly prevalent and affects a significant part of the population. Its prevalence in the general population can reach up to 5% [1], depending on the diagnostic method used. Neurophysiological staging scales for CTS already exist, one proposed by L. Padua et al. [2], and another by J.D. Bland (2000) [3].

Neurophysiologist L. Padua proposed the following criteria for differentiating moderate and severe CTS neurophysiological stages:

- Moderate: there is a slowing of median sensory conduction velocity (CV) and an abnormal median distal motor latency (DML).
- Severe: there is an unobtainable median sensory nerve action potential (SNAP) and an abnormal median DML [2].

Neurophysiologist J.D. Bland proposed similar criteria to differentiate between moderate and severe stages. He also determined a maximum value of <6.5 ms for DML to separate both of these stages from the very severe stage as follows:

- Moderate: sensory potential preserved with sensory slowing; DML to abductor pollicis brevis (APB) < 6.5 ms;
- Severe: sensory potentials absent but motor response preserved; DML to APB < 6.5 ms;
- Very severe: sensory potentials absent, but motor response preserved; DML to APB > 6.5 ms [3].

Both classifications use the absent sensory response as a main criterion to define the neurophysiological stage of severe CTS. Upon reviewing the original articles by Padua and Bland, we found that orthodromic stimulation of sensory fibers had been performed.

Nevertheless, it must be acknowledged that the antidromic potential is significantly larger and broader than the orthodromic potential at all intensities, as the sensory fibers are closer to the skin surface at the finger, where the antidromic SNAP is recorded, compared to the wrist, where the orthodromic SNAP is recorded [4]. Naturally, factors such as age, gender, body mass index, skin temperature, and impedance also influence SNAP amplitude [5,6]. Nonetheless, the depth of the nerve remains a crucial determinant, resulting in consistently smaller orthodromic SNAP amplitudes. Therefore, many laboratories (including ours in Latvia) use antidromic stimulation for sensory NCS.

However, there is no consensus on whether antidromic or orthodromic stimulation techniques should be used, and no formal guidelines for neurophysiologists have been published to date [4,7,8,9].

Through daily clinical practice, we observed that in a subset of patients, the antidromic SNAP remains detectable even when the DML surpasses 6.5 ms—a threshold proposed by Bland to distinguish the very severe stage, at which SNAP is generally absent. It is a known staging issue in Bland’s classification when there is a recordable SNAP despite a DML exceeding 6.5 ms [3].

Thus, we developed the three following hypotheses:

- A considerable number of cases exhibit a recordable antidromic SNAP, while the orthodromic SNAP is absent. Consequently, the severity of CTS, as determined by the Padua and Bland classifications, varies significantly depending on whether the orthodromic or antidromic technique is applied.
- There is an antidromic SNAP amplitude that serves as a cut-off value for undetectable orthodromic SNAP.
- There is a median nerve DML value that indicates when orthodromic SNAP registration is no longer possible, but antidromic SNAP is still present.

The aim of the study was to clarify the neurophysiological criteria for moderate and severe CTS in countries where an antidromic stimulation technique is routinely used for the registration of SNAP.

## 2. Materials and Methods

### 2.1. Equipment

For our research, we used the following tools: an EMG/NCS (Natus) workstation with disposable self-adhesive surface electrodes measuring 25 mm × 35 mm (875 mm^2^), a surface stimulation electrode, a ground electrode, a thermometer, a ruler, and alcohol wipes (for skin degreasing).

We used the thermometer for precise skin temperature establishment, as cooling results in a higher amplitude and longer duration for both compound muscle action potentials (CMAPs) and SNAPs [6]. The temperature was maintained at approximately 32 degrees Celsius (°C).

### 2.2. Patients

The study recruited patients between June and August 2024. The data were collected prospectively. Examinations were conducted at the Clinical Diagnostic Center of Riga East University Hospital in Riga, Latvia. Prior to participation, each patient signed a written consent form. Demographic data, including sex and age, were gathered. All enrolled patients exhibited clinical symptoms of CTS consistent with their reported complaints.

Inclusion criteria: patients were required to have at least a moderate stage of CTS, defined by a median nerve DML of ≥4.5 ms, and be aged 18 years or older.

Exclusion criteria: patients with coexisting polyneuropathy, traumatic median nerve injury, plexopathy, or any other peripheral nerve pathology affecting the median nerve were excluded, as were those with a history of surgically treated CTS.

### 2.3. NCS Protocol

The primary nerve examined in this study was the median nerve, with the following parameters evaluated:

Median nerve distal motor and sensory latency (ms)—for our research purposes, only onset latencies;

Median nerve CMAP and SNAP amplitude; SNAP amplitude was registered using two methods—antidromic and orthodromic stimulation;

CV of the sensory and motor fibers of the median nerve.

The ulnar nerve was used as a reference, and the same parameters were assessed.

For median and ulnar nerve motor studies, the distance between the stimulation site (SS) and the active recording electrode (G1) was 8 cm. For sensory studies of both nerves, the distance between SS and G1 was in the range of 10 cm to 15 cm (with the upper limit for median and ulnar nerves of 15 cm and 12 cm, respectively). The distance between centers of G1 and the reference electrode (G2) was 3 cm.

Median nerve stimulation using both antidromic and orthodromic techniques was conducted with an identical distance between the stimulation site (SS) and the active recording electrode (G1) in both methods.

For the antidromic stimulation technique, the stimulator was positioned on the wrist, with self-adhesive disposable electrodes placed on the third finger. In the orthodromic technique, the electrodes were applied to the wrist, while the stimulator was positioned on the third finger. The stimulation current was gradually increased until the SNAP reached its maximum amplitude. The SNAP amplitude was measured from the baseline to the peak of the negative deflection. The DSL was measured by placing a marker at the take-off point of the negative deflection. In both techniques, identical distances were used, with a minimum of 10 cm.

Once all required parameters were collected, the data were organized into a table and analyzed using IBM SPSS Statistics 25. The goal of the analysis was to find the precise cut-off values for different markers (antidromic SNAP amplitude, DML, DSL, sensory CV), that would indicate when the orthodromic SNAP amplitude would no longer be obtainable and thus signifying the severe stage of CTS. To achieve this, receiver operating characteristic (ROC) curve analysis was conducted. Orthodromic SNAP amplitude values were coded as follows: 1 for those that were recordable and 0 for those that were not. The area under the curve (AUC) was then calculated, with higher values indicating better discriminatory ability. An AUC between 0.8 and 0.9 was interpreted as excellent, and values exceeding 0.9 were considered outstanding [10].

Lastly, the Youden index was calculated for each test variable using the corresponding coordinates from the ROC curve. This calculation allowed for the identification of precise cut-off values by simultaneously optimizing both sensitivity and specificity.

## 3. Results

Data were obtained from a total of 60 hands of 47 patients with confirmed CTS and included in the analysis. Table 1 summarizes the demographic data of the study participants, most of whom were women.

The mean age of the patients was 61 years, with the youngest aged 42 and the oldest 84. Table 2 outlines the descriptive neurophysiological data for the affected hands.

**Figure 1:**
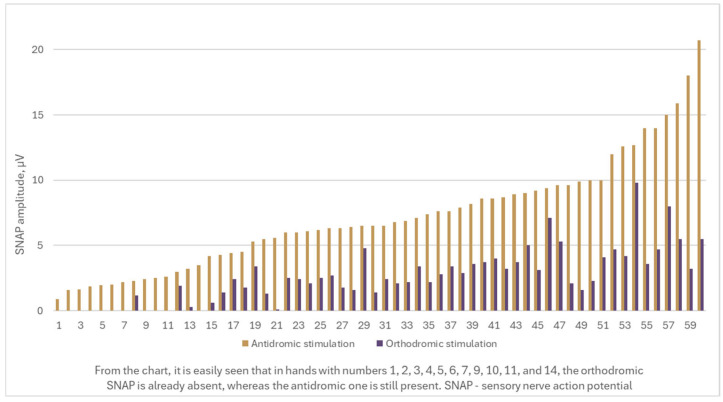
Median nerve SNAP amplitude values using antidromic and orthodromic stimulation techniques in investigated hands.

Of the total patients, 8 (13.3%) exhibited decreased amplitudes in both distal and proximal motor latencies, suggesting secondary axonal loss of motor fibers. The reference nerve, the ulnar nerve, was found to have all parameters—including SNAP and CMAP amplitudes, distal sensory and motor latencies, and sensory and motor conduction velocities—within normal ranges. The primary parameters investigated and analyzed were related to the median nerve, including DML, distal CMAP amplitude in the wrist, and all sensory fiber measurements. Importantly, orthodromic SNAP was absent in 18.3% of the examined hands, whereas the antidromic SNAP remained present (Figure 1).

ROC curve analysis was performed to determine the optimal cut-off values for predicting an undetectable orthodromic SNAP. The investigation focused on the antidromic SNAP amplitude and DML as primary predictors. Other parameters, such as antidromic DSL and sensory CV, were also analyzed for their predictive value in identifying absent orthodromic SNAP.

The analysis revealed that antidromic SNAP amplitude, DML, DSL, and sensory CV were all statistically significant markers of an undetectable orthodromic SNAP. The identified cut-off values were 3.85 (3.9) µV for antidromic SNAP amplitude, 5.14 ms for DML, 4.65 ms for DSL, and 28.05 m/s for sensory CV. The AUC values were 0.989 for antidromic SNAP amplitude, 0.920 for DML, 0.819 for DSL, and 0.803 for sensory CV. The corresponding *p*-values were <0.001 for both antidromic SNAP amplitude and DML, 0.001 for DSL, and 0.002 for sensory CV (Table 3).

The antidromic SNAP amplitude cut-off demonstrated the highest predictive accuracy, with both sensitivity and specificity values of 100% and 93.9%, respectively. The DML cut-off also showed high sensitivity (100%), but its specificity was lower at 69.4%. In comparison, both DSL and sensory CV had lower sensitivity (72.7% each) but higher specificity, at 93.9% for DSL and 87.8% for sensory CV.

Table 4 presents the differences in CTS neurophysiological staging based on the Padua and Bland classifications, depending on whether orthodromic or antidromic sensory fiber stimulation was used.

Additionally, it shows the staging results when an antidromic SNAP amplitude of <3.9 µV was applied as a cut-off to predict an absent orthodromic SNAP.

## 4. Discussion

At the time of publishing this study, we were unable to identify any previously published research comparing the use of antidromic and orthodromic stimulation techniques in patients with moderate to severe CTS. To our knowledge, this is the first study to systematically analyze sensory stimulation parameters of the median nerve and propose cut-off values for antidromic SNAP amplitude and DML to predict the absence of orthodromic responses.

The results of this study confirmed our earlier observations that antidromic SNAP remains detectable in a significant proportion of patients, even at the severe stage of CTS as defined by the Padua and Bland classifications. Specifically, 18.3% of the examined arms demonstrated absent orthodromic SNAP but retained a recordable antidromic SNAP. These findings highlight that orthodromic and antidromic techniques are not interchangeable, as relying solely on one technique could result in diagnostic errors in a considerable number of cases. Padua et al. explicitly stated that their classification system could be applied regardless of whether antidromic or orthodromic stimulation techniques were used. In contrast, Bland, who primarily used the orthodromic technique, did not offer any specific guidance on the use of antidromic methods. Our findings suggest that the antidromic technique has significant implications for accurate staging and should be considered an important component of CTS assessment.

Our proposed antidromic SNAP amplitude cut-off value for an unrecordable orthodromic SNAP demonstrated very high sensitivity and specificity. In our study, most cases with an antidromic SNAP amplitude below this threshold (3.9 µV) showed absent orthodromic SNAP responses. According to orthodromic stimulation data, these cases would be classified as severe CTS under the Padua staging system [2], and as severe or very severe under Bland’s classification [3], depending on their DML values. However, based on antidromic stimulation data, the same cases would be staged as moderate CTS under the Padua system, and as moderate or even unclassifiable under Bland’s criteria, depending on the DML value. The existing literature suggests that certain CTS cases may remain unclassifiable under Bland’s system, particularly in cases where the DML exceeds 6.5 ms but where a sensory response is still recordable. Implementing the antidromic SNAP amplitude cut-off value identified in this study as a predictor of absent orthodromic SNAP could help address this issue and reduce the number of non-classifiable cases. For neurophysiology laboratories using the Padua or Bland scales to stage CTS but applying antidromic stimulation techniques, it would be beneficial to adopt the SNAP amplitude cut-off value established in this study. However, due to potential variations in technical factors—such as signal registration parameters, recording electrode types, or equipment brands—laboratories may prefer to determine their own specific cut-off values. Additionally, examiner-related factors, such as whether the procedure is performed by a technician or a physician and the examiner’s level of experience, may also influence the results and should be considered in the development of laboratory-specific thresholds.

Our analysis revealed that a DML cut-off value was highly sensitive for predicting an absent orthodromic SNAP. While the threshold may indicate a severe stage of CTS, it cannot be used as a standalone diagnostic marker due to its low specificity.

In our study, the DSL and sensory CV cut-off values demonstrated significantly lower sensitivity and specificity compared to antidromic SNAP amplitude.

The reduced predictive value of DSL in our study may be attributed to the non-standard distance between the recording electrode and the stimulation site. However, this factor does not influence sensory CV. A slower sensory CV indicates both axonal loss in the fastest sensory fibers and more pronounced demyelination within the carpal tunnel [6]. Axonal loss directly contributes to the absence of SNAP, while demyelination leads to temporal dispersion and phase cancellation, further reducing SNAP amplitude [6]. Practically, detecting the onset of a SNAP can be challenging when the waveform is highly dispersed and of low amplitude. Several patients in our study exhibited very low-amplitude and dispersed SNAPs, making peak marker placement variable. This variability could explain the lower sensitivity and specificity of DSL and sensory CV compared to antidromic SNAP amplitude. Even if the peak DSL was used, it would likely remain insufficient to improve the predictive accuracy of these parameters for identifying advanced CTS stages.

Our study results suggest that sensory CV and onset DSL cannot be recommended as reliable diagnostic indicators for predicting an absent orthodromic SNAP.

However, it is important to recognize two selection biases present in our study.

- The first selection bias concerns the exclusion of patients with polyneuropathy. It is important to specify that this group of patients was not included in either the original Padua or Bland studies. Given the potential impact of polyneuropathy on nerve conduction parameters, this population should be studied separately. Interpreting nerve conduction results in polyneuropathic patients requires particular caution to ensure precise diagnosis.
- The second selection bias relates to the age distribution of our study participants.

## 5. Conclusions

Padua et al. suggested that both antidromic and orthodromic stimulation techniques could be used interchangeably for the staging of CTS. However, the results of our study revealed a significant discrepancy in CTS grading when the same hand was assessed using different stimulation techniques. Specifically, 18.3% of cases yielded different CTS grades depending on whether the orthodromic or antidromic recording method was employed.

We compared the AUC values for antidromic SNAP amplitude, DML, sensory CV, and DSL. Our analysis demonstrated that antidromic SNAP amplitude is the most reliable predictor of an absent orthodromic SNAP, with an AUC of 0.989. The absence of an orthodromic SNAP is indicative of severe CTS.

An antidromic SNAP amplitude cut-off value of <3.9 µV showed excellent diagnostic performance, with 100% sensitivity and 93.9% specificity, and a statistically significant *p*-value (<0.001). Based on these findings, for those applying the Padua or Bland classification systems to grade median nerve entrapment neuropathy in carpal tunnel syndrome, we recommend using this threshold to identify severe CTS cases, particularly when the DML is markedly prolonged.

Further research is warranted to validate these findings and to consider modifications to the Padua and Bland classification systems to better accommodate the antidromic stimulation technique.

## Figures and Tables

**Table 1:** Demographic data of recruited patients.

**Total number of patients, N**	47
**Sex**	
Males	17 (36.2%)
Females	30 (63.8%)
**Age**	
Mean age, years (–max)	61 (42–84)
Males’ mean age, years (min–max)	60 (44–83)
Females’ mean age, years (min–max)	61 (42–84)

**Table 2:** Nerve conduction study parameters in hands with carpal tunnel syndrome symptoms.

Nr	Sex	Age, years	DML, ms	SNAP Amplitude, µV		Onset DSL, ms	Sensory CV, m/s	CMAP Amplitude at the Distal Stimulation Site, mV	CMAP Amplitude at the Proximal Stimulation Site, mV	Motor CV, m/s
				Antidromic Stimulation	Orthodromic Stimulation					
1	male	63	5.63	4.5	1.75	3.98	29.6	4.2	3.3	55.9
2	male	61	4.81	12.6	4.2	3.73	39.7	5.3	4.9	52.0
3	male	61	4.94	12.0	4.7	3.71	38.5	5.8	5.6	51.3
4	female	63	5.14	3.5	0.0	3.04	39.5	3.2	3.1	52.1
5	male	61	7.5	1.65	0.0	4.74	27.4	0.39	0.39	31.5
6	male	61	4.60	2.3	1.18	4.17	29.9	7.0	6.7	50.9
7	female	62	4.56	5.3	3.4	4.11	35.3	6.9	6.5	50.0
8	female	64	4.71	4.2	0.61	4.21	28.5	4.6	4.1	50.0
9	male	73	4.50	8.6	3.7	4.62	39.5	8.4	7.6	50.2
10	male	73	4.57	9.4	7.1	4.96	36.0	5.9	5.4	50.3
11	female	58	6.5	9.9	1.6	4.5	27.6	6.6	6.3	62.9
12	female	60	5.9	8.6	4.0	4.3	26.8	6.3	5.0	59.5
13	female	50	4.7	8.9	3.7	3.7	34.3	4.6	4.6	57.3
14	male	61	6.1	2.6	0.0	3.8	34.2	3.2	3.2	50.0
15	female	46	4.9	9.2	3.1	3.6	33.8	9.3	9.2	51.2
16	female	46	5.2	6.9	2.2	3.8	31.3	8.3	8.1	50.0
17	female	72	5.6	6.4	1.6	4.0	31.9	4.9	4.5	54.9
18	female	72	6.5	0.9	0.0	5.3	24.5	3.2	2.9	51.1
19	female	66	4.5	8.7	3.2	3.2	38.9	8.8	7.8	59.1
20	female	47	4.9	3.0	1.9	4.6	26.55	9.7	9.0	51.3
21	male	46	6.2	3.2	0.3	5.0	26.4	7.4	7.2	60.5
22	male	46	6.8	1.6	0.0	6.2	21.0	6.5	6.2	50.0
23	male	70	6.4	2.0	0.0	6.0	21.7	4.4	4.2	50.0
24	female	65	4.6	6.5	4.8	4.2	31.4	6.0	5.2	50.0
25	female	55	5.2	9.6	5.3	3.7	32.85	6.7	6.3	51.1
26	male	62	4.7	5.5	1.3	4.2	32.5	10.1	8.9	50.0
27	female	75	5.2	6.5	1.4	4.2	27.4	6.2	5.5	50.0
28	female	42	5.4	5.6	0.1	4.3	28.5	8.6	8.6	52.3
29	male	52	4.9	6.2	2.5	3.6	36.1	5.2	4.9	57.1
30	male	44	4.8	14.0	3.6	3.8	34.0	9.2	8.7	57.3
31	male	44	5.1	7.4	2.2	4.0	31.7	10.0	8.9	57.3
32	female	54	4.65	15.9	5.5	3.8	39.2	5.8	5.5	53.8
33	female	60	5.15	1.96	0.0	4.68	26.7	2.2	1.36	53.9
34	male	55	5.28	4.4	2.4	4.20	33.3	6.9	6.9	53.8
35	female	68	6.48	2.5	0.0	4.71	27.6	4.6	4.6	58.7
36	female	68	5.52	7.6	2.8	3.78	34.4	3.8	3.8	54.6
37	male	59	4.5	10.0	2.3	3.3	39.0	6.9	6.4	55.6
38	male	56	4.6	14.0	4.7	3.6	38.9	6.2	6.0	59.5
39	male	56	5.2	7.6	3.4	4.1	34.1	5.3	5.2	55.3
40	female	78	4.6	15.0	8.0	3.3	39.4	4.7	4.3	57.1
41	female	53	5.3	6.8	2.1	4.2	31.0	4.7	4.6	54.8
42	female	66	4.5	6.5	2.4	3.4	39.0	6.7	6.5	58.8
43	male	63	4.5	9.0	5.0	3.8	36.8	5.3	5.3	57.1
44	male	55	4.51	20.7	5.5	3.00	39.4	4.3	4.3	55.3
45	male	59	4.8	18.0	3.2	3.6	38.9	8.0	7.6	55.3
46	female	78	4.5	10.0	4.1	3.2	38.2	6.3	6.0	55.6
47	female	78	4.8	7.9	2.9	3.4	38.2	4.5	4.0	57.1
48	male	83	4.6	6.3	2.7	3.6	38.9	3.7	3.7	46.7
49	female	78	5.3	2.2	0.0	4.2	33.3	5.0	5.0	54.5
50	female	65	5.2	6.0	2.5	4.5	31.1	5.2	5.2	58.5
51	female	84	5.1	4.3	1.4	4.0	29.8	7.1	6.6	52.6
52	female	73	7.06	2.4	0.0	5.19	25.0	6.4	6.3	58.2
53	female	73	6.98	1.85	0.0	5.11	25.4	6.3	6.0	61.5
54	female	60	4.60	12.7	9.8	3.21	38.9	7.6	7.1	59.4
55	female	60	5.99	6.0	2.4	4.43	27.1	4.3	4.1	50.6
56	female	52	5.07	6.1	2.1	3.96	31.6	5.1	4.9	59.2
57	female	48	4.59	9.6	2.1	3.84	36.45	4.0	3.8	59.1
58	female	51	4.50	8.2	3.6	3.87	36.1	5.0	5.0	58.5
59	female	50	5.13	6.3	1.78	4.69	29.9	5.5	5.2	57.5
60	female	50	5.05	7.1	3.4	3.92	35.75	2.8	2.8	52.6

DML—distal motor latency. SNAP—sensory nerve action potential. DSL—distal sensory latency. CV—conduction velocity. CMAP—compound muscle action potential.

**Table 3:** ROC curve analysis for antidromic SNAP, DML, DSL, and sensory CV parameters to define precise cut-off value, when orthodromic SNAP amplitude value equals zero.

Variables	AUC	Standard Error	*p* Value	Confidence Interval		Youden Index	Optimal Cut-Off Value	Sensitivity
				Lower Bound	Upper Bound			
Antidromic SNAP amplitude	0.989	0.010	<0.001	0.969	1.000	0.939	3.9 µV	100%
DML	0.920	0.041	<0.001	0.840	1.000	0.694	5.14 ms	100%
DSL	0.819	0.094	0.001	0.635	1.000	0.666	4.65 ms	72.7%
Sensory CV	0.803	0.093	0.002	0.621	0.985	0.605	28.05 m/s	72.7%

AUC—area under the curve. SNAP—sensory nerve action potential. DML—distal motor latency. DSL—antidromic distal sensory latency. CV—conduction velocity.

**Table 4:** CTS cases’ staging by Padua or Bland using different sensory fibers’ stimulation techniques.

Investigated Arms, Nr	DML, ms	SNAP Amplitude, µV		CTS Staging Using Orthodromic Stimulation	CTS Staging Using Antidromic Stimulation	CTS Staging Using Antidromic SNAP Amplitude Cut-Off Value of ≤3.85 µV
		Orthodromic Stimulation	Antidromic Stimulation			
1	5.63	1.75	4.5	moderate	moderate	moderate
2	4.81	4.2	12.6	moderate	moderate	moderate
3	4.94	4.7	12.0	moderate	moderate	moderate
4	5.14	0.0	3.5	**severe**	**moderate**	**severe**
5	7.5	0.0	1.65	**severe (very severe) ^**	**moderate (cannot classify) ^**	**severe (very severe) ^**
6	4.60	1.18	2.3	*moderate*	*moderate*	*severe*
7	4.56	3.4	5.3	moderate	moderate	moderate
8	4.71	0.61	4.2	moderate	moderate	moderate
9	4.50	3.7	8.6	moderate	moderate	moderate
10	4.57	7.1	9.4	moderate	moderate	moderate
11	6.5	1.6	9.9	moderate	moderate	moderate
12	5.9	4.0	8.6	moderate	moderate	moderate
13	4.7	3.7	8.9	moderate	moderate	moderate
14	6.1	0.0	2.6	**severe**	**moderate**	**severe**
15	4.9	3.1	9.2	moderate	moderate	moderate
16	5.2	2.2	6.9	moderate	moderate	moderate
17	5.6	1.6	6.4	moderate	moderate	moderate
18	6.5	0.0	0.9	**severe**	**moderate**	**severe**
19	4.5	3.2	8.7	moderate	moderate	moderate
20	4.9	1.9	3.0	*moderate*	*moderate*	*severe*
21	6.2	0.3	3.2	*moderate*	*moderate*	*severe*
22	6.8	0.0	1.6	**severe (very severe) ^**	**moderate (cannot classify)**	**severe (very severe)**
23	6.4	0.0	2.0	**severe**	**moderate**	**severe**
24	4.6	4.8	6.5	moderate	moderate	moderate
25	5.2	5.3	9.6	moderate	moderate	moderate
26	4.7	1.3	5.5	moderate	moderate	moderate
27	5.2	1.4	6.5	moderate	moderate	moderate
28	5.4	0.1	5.6	moderate	moderate	moderate
29	4.9	2.5	6.2	moderate	moderate	moderate
30	4.8	3.6	14.0	moderate	moderate	moderate
31	5.1	2.2	7.4	moderate	moderate	moderate
32	4.65	5.5	15.9	moderate	moderate	moderate
33	5.15	0.0	1.96	**severe**	**moderate**	**severe**
34	5.28	2.4	4.4	moderate	moderate	moderate
35	6.48	0.0	2.5	**severe**	**moderate**	**severe**
36	5.52	2.8	7.6	moderate	moderate	moderate
37	4.5	2.3	10.0	moderate	moderate	moderate
38	4.6	4.7	14.0	moderate	moderate	moderate
39	5.2	3.4	7.6	moderate	moderate	moderate
40	4.6	8.0	15.0	moderate	moderate	moderate
41	5.3	2.1	6.8	moderate	moderate	moderate
42	4.5	2.4	6.5	moderate	moderate	moderate
43	4.5	5.0	9.0	moderate	moderate	moderate
44	4.51	5.5	20.7	moderate	moderate	moderate
45	4.8	3.2	18.0	moderate	moderate	moderate
46	4.5	4.1	10.0	moderate	moderate	moderate
47	4.8	2.9	7.9	moderate	moderate	moderate
48	4.6	2.7	6.3	moderate	moderate	moderate
49	5.3	0.0	2.2	**severe**	**moderate**	**severe**
50	5.2	2.5	6.0	moderate	moderate	moderate
51	5.1	1.4	4.3	moderate	moderate	moderate
52	7.06	0.0	2.4	**severe (very severe) ^**	**moderate (cannot classify) ^**	**severe (very severe) ^**
53	6.98	0.0	1.85	**severe (very severe) ^**	**moderate (cannot classify) ^**	**severe (very severe) ^**
54	4.60	9.8	12.7	moderate	moderate	moderate
55	5.99	2.4	6.0	moderate	moderate	moderate
56	5.07	2.1	6.1	moderate	moderate	moderate
57	4.59	2.1	9.6	moderate	moderate	moderate
58	4.50	3.6	8.2	moderate	moderate	moderate
59	5.13	1.78	6.3	moderate	moderate	moderate
60	5.05	3.4	7.1	moderate	moderate	moderate

CTS—carpal tunnel syndrome. SNAP—sensory nerve action potential. DML—distal motor latency. ^—CTS stage by Bland is given in brackets in cases where it differs from stage by Padua. Stages in **bold**—in these cases, the stage changes depending on the used sensory diagnostic method (orthodromic vs. antidromic). Stages in *italic*—in these cases, the stage changes if our proposed sensory antidromic cut-off value is used. According to Bland classification, there is no clear recommendation on how one should stage cases, where SNAP is present, but DML is >6.5. Therefore, we marked them as “can’t classify”.

## Data Availability

Data are contained within the article.

## Abbreviations

The following abbreviations are used in this manuscript:

AUC	area under the curve
CMAP	compound muscle action potential
CTS	carpal tunnel syndrome
CV	conduction velocity
DML	distal motor latency
DSL	distal sensory latency
G1	active recording electrode
G2	reference electrode
NCS	nerve conduction study
ROC	receiver operating characteristic
SNAP	sensory nerve action potential
SS	stimulation site
